# Supplementary material for: Independent control of amplitude and period in a synthetic oscillator circuit with modified repressilator
Source: Commun Biol. 2022 Jan 11;5:23. doi: 10.1038/s42003-021-02987-1 (PMC8752629; doi:10.1038/s42003-021-02987-1)
Supplement: Supplementary file 2 — Supplementary Information [file 42003_2021_2987_MOESM2_ESM.pdf]

Supporting information

Independent control of amplitude and period in a synthetic oscillator circuit with modified repressilator

Fengyu Zhang[1,2], Yanhong Sun[2], Yihao Zhang[1,3], Wenting Shen[2,3], Shujing Wang[2,3], Qi Ouyang[3], Chunxiong Luo[2,3,4,5]*

[1]School of Life Sciences and Peking-Tsinghua Center for Life Sciences, Peking University, Beijing 100871, China.
[2]The State Key Laboratory for Artificial Microstructures and Mesoscopic Physics, School of Physics, Peking University, China;
[3]Center for Quantitative Biology, Academy for Advanced Interdisciplinary Studies, Peking University, China.
[4]Wenzhou Institute University of Chinese Academy of Sciences, Wenzhou, Zhejiang, China;
[5]Oujiang Laboratory, Wenzhou, Zhejiang, China

*To whom correspondence should be addressed: Chunxiong Luo, email: pkuluocx@pku.edu.cn.

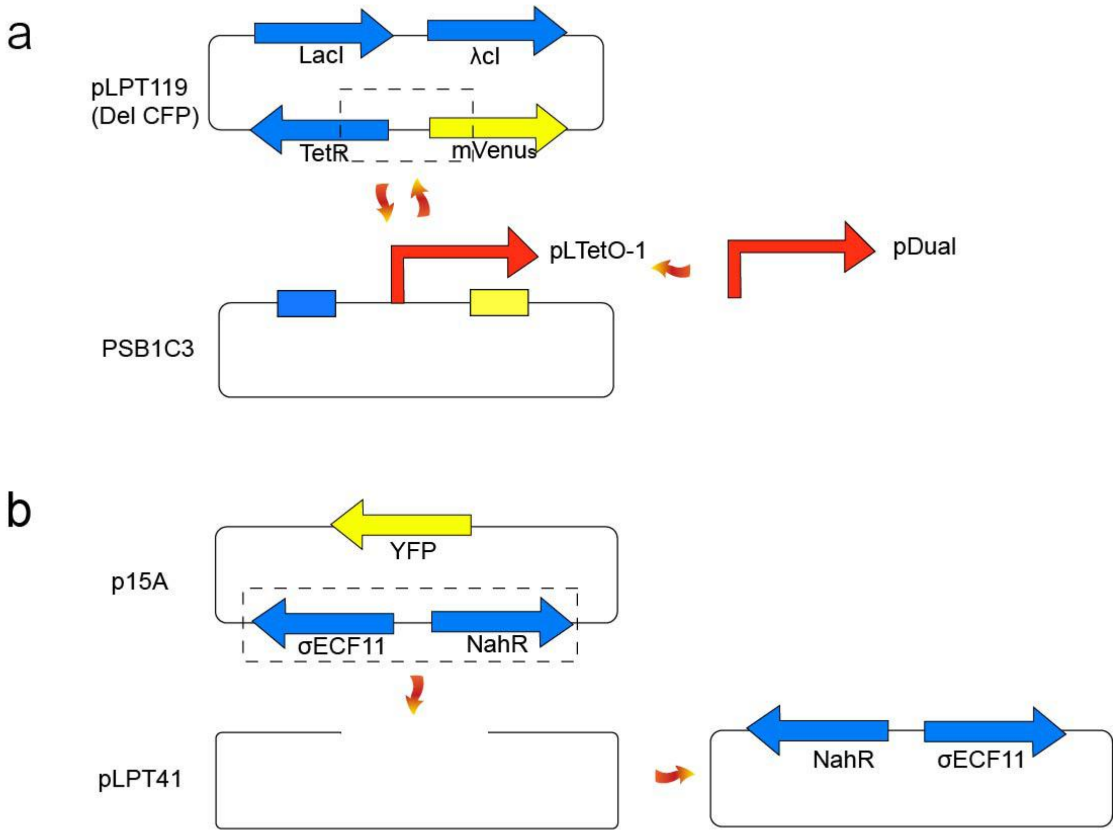

Supplementary Figure 1. Detailed cloning steps of the repressilator reconstruction. **a.** The reconstruction of pLPT119 plasmid. The original pLTetO-1 promoter of the mVenus gene was changed to our pDual promoter in a subcloning plasmid with the PSB1C3 vector. Then the fragment containing the pDual promoter was reinsert to pLPT119 plasmid by Gibson assembly. **b,** The reconstruction of pLPT41 plasmid. The *σECF11* gene, the *NahR* gene and relevant promoters were separated from a p15A plasmid and then integrated into the pLPT41 plasmid by Gibson assembly.

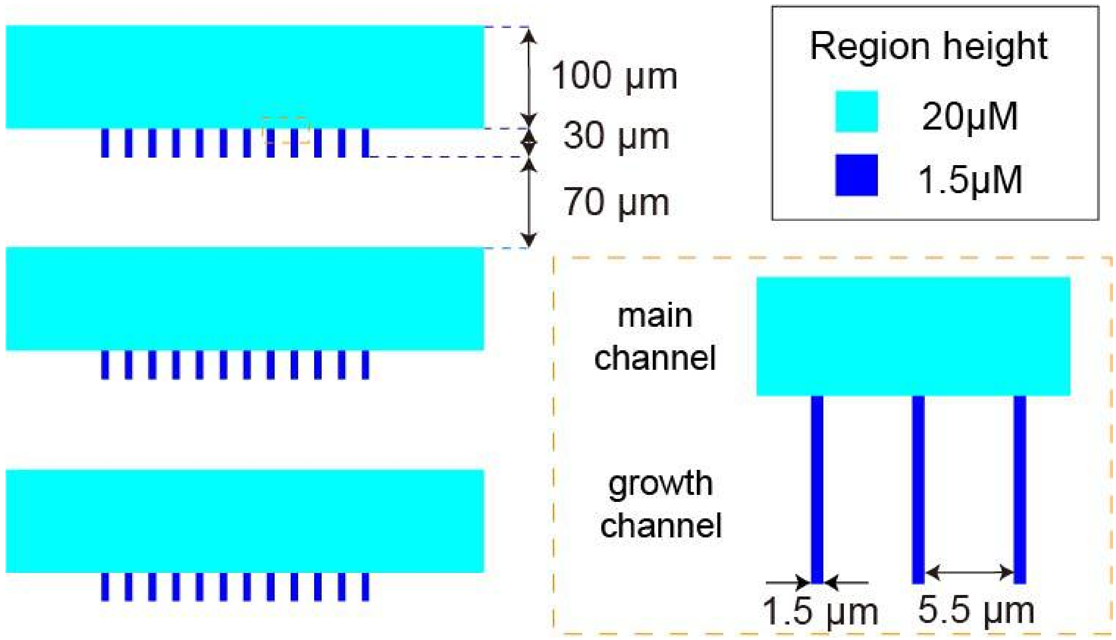

Supplementary Figure 2. Details of microfluidic chips design. The main channels were designed to be 100μm width and 20μm height. The growth channels were designed to be about 1.5μm width, 1.5μm height and 30μm in length.

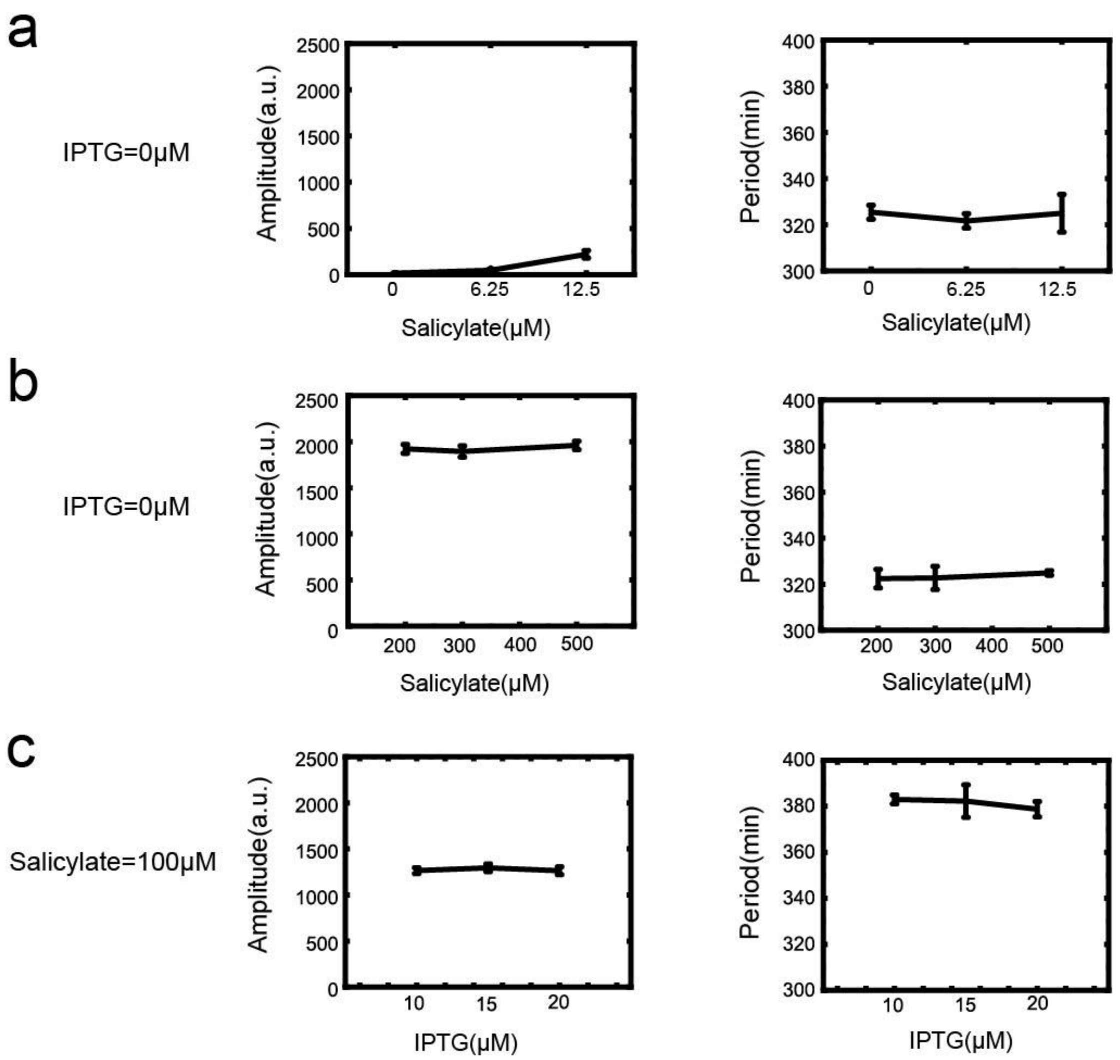

Supplementary Figure 3. The limitation of the orthogonal regulation of amplitude and period. **a.** The amplitude of the oscillation when the salicylate concentration was under 50 μM. The regulation had already worked at 6.25 μM. The concentration of IPTG was set to 0 μM. **b.**The amplitude of the oscillation when the salicylate concentration was greater than 200 μM. There is no significant increase in 300 μM and 500 μM groups. The concentration of IPTG was set to 0 μM. **c.** The period of the oscillation when the IPTG concentration was greater than 10 μM. There is no significant increase in 15 μM and 20 μM groups. The concentration of salicylate was set to 100 μM. The data point were calculated from the average of the three groups of results and the error bars were calculated from the SD of the average of them.

Name	Model Name	Initial Amount	Units
λcI	R_1	0	Molecules
LacI	R_2	0	Molecules
TetR	R_3	100	Molecules
NahR	R_4	0	Molecules
σECF11	E	0	Molecules
mVenus	Y	0	Molecules
λcI mRNA	m_{R1}	0	Molecules
LacI mRNA	m_{R2}	0	Molecules
TetR mRNA	m_{R3}	0	Molecules
NahR mRNA	m_{R4}	0	Molecules
σECF11 mRNA	m_E	0	Molecules
mVenus mRNA	m_Y	0	Molecules
Salicylate	I_1	0/50/100/200	μM
IPTG	I_2	0/2.5/5/10	μM
Unfolded λcI	R_{1u}	0	Molecules
Unfolded LacI	R_{2u}	0	Molecules
Unfolded TetR	R_{3u}	0	Molecules
Unfolded NahR	R_{4u}	0	Molecules
Unfolded σECF11	E_u	0	Molecules
Unfolded mVenus	Y_u	0	Molecules

Supplemental Table 1. Model species

Description	Name	Value	Units
λcI copy nuber	p_1	1	Molecules
LacI copy number	P_2	1	Molecules
TetR copy number	P_3	1	Molecules
NahR copy number	P_4	1	Molecules
σECF11 copy number	p_e	1	Molecules
mVenus copy number	p_y	1	Molecules
λcI basal expression rate	b_1	0.0012	min^-1
LacI basal expression rate	b_2	0.0012	min^-1
TetR basal expression rate	b_3	0.0012	min^-1
NahR basal expression rate	b_4	0.0012	min^-1
σECF11 basal expression rate	b_e	0.0012	min^-1
mVenus basal expression rate	b_y	0.0012	min^-1
λcI maximum expression rate	a_1	10	min^-1
LacI maximum expression rate	a_2	10	min^-1
TetR maximum expression rate	a_3	10	min^-1
NahR maximum expression rate	a_4	10	min^-1
σECF11 maximum expression rate	a_e	10	min^-1
mVenus maximum expression rate	a_y	20	min^-1
λcI translation rate	r_1	50	min^-1
LacI translation rate	r_2	50	min^-1
TetR translation rate	r_3	50	min^-1
NahR translation rate	r_4	50	min^-1
σECF11 translation rate	r_e	50	min^-1
mVenus translation rate	r_y	50	min^-1
λcI folding rate	k_{f1}	0.4	min^-1
LacI folding rate	k_{f2}	0.4	min^-1
TetR folding rate	k_{f3}	0.4	min^-1
NahR folding rate	k_{f4}	0.4	min^-1
σECF11 folding rate	k_{fe}	0.4	min^-1
mVenus folding rate	k_{fy}	0.4	min^-1
λcI character concentration	k_1	5.5	Molecules
LacI character concentration	k_2	1	Molecules
TetR character concentration	k_3	3	Molecules
NahR character concentration	k_4	2.3	Molecules
σECF11 character concentration	k_e	13.5	Molecules
Salicylate character concentration	k_{I1}	4	μM
IPTG character concentration	I_0	0.5	μM
mRNA degradation rate	μ	0.35	min^-1
Protein dilution rate	δ	0.015	min^-1

Supplemental Table 2. Model Parameters

Supplementary Note 1. Detailed cloning steps

On the basis of our design strategy to independent control of amplitude and period of repressilator, we developed a gene circuit construction from the pLPT119 and pLPT41 plasmids used in Potvin`s work[10]. The original pLPT119 plasmid had the original repressilator and an *mVenus* fluorescent protein gene which act as a reporter. It also had a constant-expressed *CFP* gene as a marker, which was deleted in our subsequent design. The original pLPT41 plasmid only had no functional genes except two binding sites of TetR.

To change the promoter of the reporter gene, *mVenus*, we constructed a subcloning containing the upstream and downstream gene sequence of the original promoter, which was a pLTetO-1 promoter. Then the dual promoter was added into the subcloning by repeatedly PCR steps. Then the subcloning was integrated into the original pLPT119 plasmid by Gibson assembly.

The control system of σECF11 was introduced into the pLPT41 plasmid. The *σECF11* gene was designed to be regulated by an *NahR-pSal* system[16][17]. The *σECF11* gene, the *NahR* gene and relevant promoters were all constructed into the pLPT41 plasmid by Gibson assembly.
