# Supplementary material for: Independent control of amplitude and period in a synthetic oscillator circuit with modified repressilator
Source: Commun Biol. 2022 Jan 11;5:23. doi: 10.1038/s42003-021-02987-1 (PMC8752629; doi:10.1038/s42003-021-02987-1)
Supplement: Supplementary file 3 — Description of Additional Supplementary Files [file 42003_2021_2987_MOESM3_ESM.pdf]

Description of Additional Supplementary Files

File name: Supplementary Data 1
Description: The sequences of biological parts used in this study.
